# Customized Manual Muscle Testing for Post-Stroke Upper Extremity Assessment

**DOI:** 10.3390/brainsci12040457

**Published:** 2022-03-28

**Authors:** Nadinne Alexandra Roman, Roxana Steliana Miclaus, Cristina Nicolau, Gabriela Sechel

**Affiliations:** 1 Faculty of Medicine, Transilvania University of Brasov, 500036 Brasov, Romania; nadinneroman@unitbv.ro (N.A.R.); gabisechel@yahoo.com (G.S.); 2 Faculty of Economic Sciences and Business Administration, Transilvania University of Brasov, 500036 Brasov, Romania; cristina.nicolau@unitbv.ro

**Keywords:** assessment, muscle strength, post-stroke rehabilitation, upper extremity

## Abstract

In neuro-rehabilitation, the assessment of post-stroke patients’ motor function of damaged upper extremities (UEs) is essential. Clinicians need clear and concise assessment instruments to monitor progress recorded in intensive rehabilitation sessions. One such instrument is Manual Muscle Testing (MMT), which, in our view, requires a modified scoring model aimed at improving the assessment process of patients’ motor and functional UE status, and recording their step-by-step-progress, especially if patients undergo a short length of hospitalization (of about 10 therapy days). Hence, this paper presents a new scoring system developed by the authors. This systemresults in a more precise MMT grading scale, which has more grades and can provide a more specific muscular assessment, while offering more clarity in quantifying patients’ progress after physical therapy. A prospective study was made of 41 post-stroke patients with upper extremity (UE) impairments. To determine the validity of the assessment tool for hypothesizing, and the unidimensionality and internal consistency of the customized model, exploratory and confirmatory factor analysis (CFA) with a structural equation model (SEM), Cronbach’s Alpha, and Pearson correlation coefficients were used with Fugl–Meyer (FM) assessments, the Modified Ashworth Scale (MAS), AROM, and the Modified Rankin Scale (MRS). Considering the unidimensionality of the instrument used, we performed a linear regression to identify whether certain movements performed segmentally by the manually evaluated muscles influence the measured manual score of the whole UE. All indices suggested a good model fit, and a Cronbach’s Alpha of 0.920 suggested strong internal consistency. The Pearson correlation coefficient of the MMT-customized score with AROM was 0.857, *p* < 0.001; that with FMUE was 0.905, *p* < 0.001; that with MRS was −0.608, *p* = 0.010; and that with MAS was −0.677, *p* < 0.001. The linear regression results suggest that wrist extensors, shoulder abductors, and finger flexors can influence the manual assessment of the muscle strength of the whole UE, thereby improving post-stroke patient management. The results of our research suggest that, using the proposed scoring, MMT may be a useful tool for UE assessment in post-stroke patients.

## 1. Introduction

### 1.1. Functional Diagnosis of Post-Stroke Patients’ Upper Extremities

The assessment and functional diagnosis of patients represent the first step of their rehabilitation process. Evaluation helps medical practitioners to set the rehabilitation goals and to outline the physical therapy programs.

The use of standard methods or scientifically validated evaluation scales can be invaluable in maximizing the details of data collection and in reducing the time for diagnosis and goal setting. In addition to establishing the elements related to disability, the evaluation in medical rehabilitation and physiotherapy includes objective and subjective evaluation modalities that allow the clinical and functional diagnoses to be achieved. Functional assessment and diagnosis are related to daily activities (ADLs), instrumental daily activities (I-ADLs), or professional activities [1,2,3].

The most common impairments in the acute and chronic stages of post-stroke are cognitive states and motor deficits contralateral to the affected cerebral hemisphere. After stroke, there is profound neuromuscular reorganization. Depending on the brain injury site and dimensions, the affected limb either loses muscle strength or is characterized by spasticity, abnormal synergic motions with stereotyped movement patterns, caused mainly by abnormal co-activation of muscles and increased activity of antagonistic muscles [4,5].

Currently, patients are assessed mainly by clinical scales, with the Fugl–Meyer test being one of the most commonly used measures of motor impairment after stroke [6]. However, the accuracy of these clinical tests is limited by inter-rater and intra-rater reliability, and floor and ceiling effects. In addition, some of them require a considerable amount of time to perform. Clinical scales should therefore be combined with targeted neuro-biomechanical assessments to provide a more detailed description of the patient’s clinical condition [3].

The functional diagnosis allows the medical practitioners to set the post-stroke patients’ rehabilitation objectives, and can also provide a perspective on the future rehabilitation potential and prognosis, and on the necessary timeframe [7]. In stroke, spontaneous neuromotor recovery is known to occur approximately three months after the incident, so the ability to recover from severe neuromotor impairment differs from patient to patient, depending on the type of stroke, the capacity of spontaneous neurorehabilitation, early treatment, and early application of the rehabilitation protocol. There is a big difference in terms of evolution and prognosis between a patient who had a recent episode of stroke and is referred to rehabilitation in the sub-acute phase, and a patient who had a stroke two years ago, who is already in the chronic phase and has not benefited from early rehabilitation [8,9,10].

The medical history and physical examination of post-stroke patients, in addition to the continuous updating of the literature in the field of UE assessment and rehabilitation by physicians, represent added medical value. Medical rehabilitation and physical therapy aim to provide safe, efficient, and high-quality care and rehabilitation services to improve the health and function of individuals. Therefore, the use of evidence-based treatments, performance scales, and globally defined standards in physical medicine is critical to the development of a valuable and robust healthcare system [11] that is patient centered.

When used appropriately by clinicians who have the necessary skills, validated measurement tools, even in adapted forms, are part of the improvement process of assessment and diagnosis in the context of rehabilitation development. In clinical research, the greatest advantage is that they provide clinicians and researchers with data and meaningful indicators for clinical practice and decision making [12,13].

### 1.2. Manual Muscle Testing Scoring System and Its Patient-Customized Variations

Manual testing of muscles is performed with the hands of the therapist or physician, isokinetic machines, and other portable devices. However, isokinetic machines and dynamometers used for more objective muscle tests are still too expensive or burdensome for clinical use, although these devices are valuable for research purposes [14,15]. The Manual Muscle Testing (MMT) scoring system is an assessment tool used by rehabilitation physicians or physiatrists, physiotherapists, neurologists, and other clinicians who deal with the individuals’ functional status. The most frequently used approach is the use of MMT to assess the grade of muscle weakness in different pathologies [16]. To date, muscle strength has been assessed using Wright and Lovett’s classical system developed in 1912, or with customized variants, such as:

(a) the Medical Research Council (MRC), scale which uses a numerical grading similar to the classical MMT scale, but differs from it because the 4th and the 5th forces are defined differently [15,17];
(b) the Kendall Scale, which uses a percentage gradation of the muscle strength and assesses individual muscles [14];
(c) Daniel and Worthingham’s scale, which uses a five-point scale defined as normal, good, fair, poor, trace and zero, and assesses muscles that perform a joint motion, rather than individual muscles [15];
(d) Noureau and Vachon’s scale [18], which comprises a more systematized notation variant, through which the differentiated distinction of the degrees of muscular strength may be made. The authors propose a grading system from 0 to 5, with 0.5-point splitting [18].

The classical MMT scoring system, also known as the Oxford Scale or Medical Research Council Manual Muscle Testing scale [15], is a six-point assessment system, as described in Table 1. Its development is attributed to Wright and Lovett [19]. It was first used to assess muscle strength impairments manifested during the polio outbreak in the early part of the 20th century, which are related to progressive muscle weakness followed by muscle atrophy, fibrosis and retraction, pain from joint degeneration, and fatigue [19].

In 1940, Kendall and Kendall [14] improved the assessment technique by establishing specific testing positions and the “breaktest”. The Manual Muscle Testing taught today incorporates Wright and Lovett’s antigravity testing methods, with Kendall’s refinement. Kendall points out that the examiner’s skill is paramount in accurately assessing muscle strength. Traces of muscle contractions (grade 1) are assessed by comparison with the lack of muscle contraction (grade 0) based on examiner’s visual inspection and palpation skills. Grade 2, weak muscle contraction, differs from grade 3 by position, grading two without the involvement of gravity, while for grading three the motion is performed against gravity; both scoring points require complete movement. However, this common clinical method of assessing muscle strength has limitations, such as poor sensitivity and diagnostic accuracy of only 78% [20] compared to technological measurement systems, for example, dynamometry [21].

Since 1940, different adaptations or customizations of MMT scoring have been made, especially in the last two decades, responding to the needs of patients with different muscle impairments. Adaptations of the MMT score use +/− after every score to allow a more complex appreciation ofthe developed muscle force.

A notable modified MMT scoring scale was proposed by Noureau and Vachon in 1999 [18] in their research focused onspinal cord injuries. Depending on the amplitude of motion performed by the subject, their approach split the scoring intervals into two, resulting in four additional scores: 1.5, 2.5, 3.5, and 4.5. The same scoring protocol was used in children with Spina Bifida, in 2009, by Mahony et al. [22]. Both attempts showed good reliability and a robust assessment correlation with dynamometry as an objective evaluation method.

MMT has become an assessment tool in all rehabilitation fields. However, it has higher importance in neurological pathologies because it actually assesses the ability of the muscle to respond adaptively, to generate muscle response by recruiting motor units, to “reply” to the resistance of the therapist, and to keep the mobilized segment at the end of ROM. For grade five, the resistance provided by the therapist can be submaximal resistance (“make-test”) or maximal resistance (“break-test”). Clinical practice highlighted that there were differences among the different specialists’ assessments, caused either by the use of less rigorous techniques, by the time spent on the make test or the break test, or by the stabilization and positioning of the patients. Therefore, a rigorous protocol must be used and respected in each MMT assessment setting [16,20,23,24].

Unlike the assessment of muscle weakness in intact nervous system pathologies, in neurological rehabilitation, especially within short-length hospital stays, assessment precision is needed. Within our activity, we observed that for the post-stroke patient, who most frequently suffers from spasticity, stiffness, retraction, and fibrosis of conjunctive tissue, special attention is necessary when performing any assessment, which should be correct, technically impeccable, rigorous, repetitive, and frequent (sometimes every 3–4 days). For these reasons, every patient’s practical assessment, and the outlining of an effective individual physical therapy program, may be time consuming. Another essential feature of differences between MMT assessment in different pathologies is related to the make and break tests. For the break test to be accurately performed, in some motions, the examiner cannot provide enough resistance to generate an eccentric muscle contraction response. However, in neurorehabilitation, especially in post-stroke patients, when MMT can be performed bilaterally, the make test is sufficient for the detection of neuromuscular adaptation of the opposing resistance at a fixed point. Our study focused on the neurological impairment manual muscle assessment, specifically using the make test, because the practical procedure clearly differs in the form of MMT; in some cases, the patient needs to resist a fixed resistance, whereas, in others, the patient has to react and adapt to the pressure applied progressively by the tester [16].

Under such circumstances, this paper presents a new approach that more efficiently uses the MMT scale by customizing it for stroke patients. In our view, stroke patients need a more specific muscular assessment. A more precise MMT grading scale having more grades would meet this need, in addition to offering more clarity in quantifying patients’ progress during the physical therapy.

### 1.3. Customizing the Manual Muscle Testing Scoring System for Post-Stroke Patients

With regard to post-stroke patients, three categories of factors justify the need to adapt the MMT scoring system:

- Patient-related: When using the MMT scoring scale for patients with stroke or significant neuromotor dysfunctions, a constraint of the classical MMT is that muscle force is evaluated on an ordinal scale rather than a continuous scale [21]. Thus, subtle variations in muscle strength are challenging to identify, especially because, in neuro-rehabilitation, the recovery process is longer than that in other rehabilitation areas (musculoskeletal, post trauma etc.). These issues are of primary clinical importance because although small changes in post-stroke muscle force may not be evident on the MMT scale, slight improvements in muscle strength may be enough to adjust the training programs or the goals set for stroke patients [15,21].
- Examiner-related: The results of the MMT scale depends on the examiner’s skills to provide external resistance to the motion performed by the patient, especially with regard to the 4th and 5th grades, at least in regard to Daniel and Worthingham’s testing [15]. In addition to the resistance provided by gravity to the weight of the mobilized segment, the therapist tries to bring the segment into the initial position after the movement has been performed for the complete amplitude, with an added resistance of a moderate type (defined as the 4th grade) or with a maximum resistance or a breaktest (defined as the 5th grade/‘normal’/’good’ depending on the variant used). Moreover, the assessment may fluctuate, if in a clinical examination or study, there are more examiners working with different patients. Previous studies highlight that, due to MMT variability, the results of the inter-rater reliability values are disputable [20,23]. Finally, it can be challenging for inexperienced physicians or physiotherapists (with 5 to 10 years of experience) to distinguish subtle fluctuations in muscle force when using MMT [24].
- Scoring-related: Since significant differences may be considered in the six-point scale of the MMT system, its guidelines suggest using the symbols ‘+/−‘before or after the scoring numbers so as to give a more accurate framework for assessing muscle strength [25]. In addition to the MMT scoring differences, another important reasoning for customizing the MMT scale for post-stroke patients is the technique used in muscle strength evaluation. The Kendall technique implies individual muscle assessment and, at present, its beneficial didactic and instructional value is preserved. However, it cannot be used in clinical programs, which need the assessment of synergistic muscle groups that perform movements on different motion axes. Daniel and Worthingham’s technique is also important in practice. It assesses muscle groups that mobilize a segment in a specific analytical direction of movement, and this way, it allows a more functional assessment that relates to directions of movement and muscle groups than is the case for a specific muscle. Therefore, the required assessment time is shorter, and, at the same time, important information is provided about the patient’s muscle strength. In neuro-rehabilitation, physical therapy programs focus on relearning functional movements by counteracting abnormal movement patterns characteristic of the post-stroke condition. Therefore, an assessment of muscle groups is more appropriate for establishing a functional diagnosis and setting goals [15]. Our research proposes a modified scoring for the MMT system that is customized for post-stroke patients, based on the active range of motion degrees, specific to each analytical motion and muscle group that performs the movement. We hypothesized that if we scored MMT based on a range of motion degrees that was divided into four sections, we could obtain more specific and accurate MMT values in post-stroke patients, which may be successfully used by clinicians and researchers. Moreover, by assessing the group muscles involved in unidirectional joint movement, the time used for the assessment would be reduced compared to a single muscle assessment (responding to an essential need of the human resources implied in the clinical assessment). Patients’ fatigue, which is a limiting factor of their physical activity and movement, would also be reduced. Therefore, our primary outcome was to identify if the adapted MMT scoring system was a reliable and valid tool in post-stroke assessment, and secondarily, to identify the muscle groups’ inference in the UE functionality for performing ADLs and thus predict the limb’s rehabilitation.

Thus, we hypothesized that a better framework can be obtained for the assessment, functional training, and outcomes in post-stroke patients.

We also aimed to identify if this scoring method correlated with maximum ROM as a reference point, if it can highlight small changes in muscle strength in chronic post-stroke patients during 10 days of neuro-rehabilitation, and if it can determine if this type of assessment might be a tool with unidimensional construct validity and internal consistency.

## 2. Materials and Methods

### 2.1. Study Localization and Ethical Issues

The study was performed at the Clinical Hospital of Psychiatry and Neurology in Brașov, Romania, from July 2019 to July 2021, as a part of more extensive research regarding physical therapy in post-stroke patients. The study was registered in clinicaltrials.gov under no. NCT04436770. The Research and Ethics Committee of the Clinical Hospital of Psychiatry and Neurology in Brașov approved the research, no. 12534/18 July 2019. Every patient included in the research freely consented to research participation. According to the European legislation, all the patients were informed regarding the chance to withdraw from the study at any time. Informed consent was obtained, and no personal data were used in this research.

Results of previous extensive research regarding physical therapy in post-stroke patients revealed the superior benefits of virtual reality (VR) as a modern treatment method versus standard kinetic therapy. During this extensive study, an additional objective was defined, specifically, the need to customize the scales and classical methods of assessment, such as MMT in neurological patients, for a more accurate and high-quality assessment, also to record patient’s motor and functional progress.

### 2.2. Participants

A total of 48 patients were enrolled in the research. Every participant of the sample had to meet the following inclusion criteria:

(a) being a chronic survivor of a stroke that occurred more than six months and less than four years earlier;
(b) not suffering from severe cognitive impairments, global or transcortical sensory aphasia, anemia, atrial fibrillation, or NYHA class IV heart failure;
(c) having no other injury or dysfunction of the UE, such as fractures, periarthritis, or moderate-severe pain;
(d) being able to perform at least 30-degree flexion and scapulohumeral abduction against gravity and at least 30-degree elbow flexion against gravity.

Thus, the sample was reduced to 41 patients, because 7 enrolled patients developed anemia or atrial fibrillation during hospital admission. Table 2 shows the patient’s age, gender, time since stroke, and the body side affected by hemiparesis.

Another component of the characteristics of the studied group is linked with the mean values regarding the initial assessments of the Functional Independence Measure (FIM), with a mean of 116.67 (SD 18.37); the Fugl–Meyer Upper Extremity Assessment (FMUE), with a mean of 46.10 (SD13.13); the Modified Rankin Scale (MRS), with a mean value of 1.9 (SD 0.77; and the Modified Ashworth Scale (MAS), with an initial assessment of 1.65 (SD 0.86). Therefore, the participants from our research can be considered as post-stroke survivors having mild impairments.

### 2.3. Outcome Measures

The research methodology required that patients’ UEs were assessed by four experienced physiotherapists, before and two weeks after the intervention, with the following scales: FIM, MRS, MAS, FMUE, Manual Muscle Testing (MMT) for muscle strength, and Active Range of Motion (AROM) for range of motion. The characteristics of the outcome measures and their roles are depicted in Table 3.

### 2.4. Procedures

The patients were assessed in the first day of hospitalization and at discharge, after 10 treatment days. The research used the test–intervention–test methodology [36]. The subjects received 60 min sessions of standard physical therapy, for the UE and hand dexterity. Physical therapy exercises included analytical passive, self-mobilization, and active mobilization of the UE segments (shoulder, elbow, forearm, wrist, fingers), UE Kabat diagonals, strength exercises for the antagonist muscles (opposite to spastic muscles), and functional tasks of daily living. The dexterity exercises included the Canadian plate use, mirror therapy for patients with muscle strength ≤1 (for wrist and finger motions because they could not perform tasks on the Canadian plate or dexterity exercises), the functional exercises of grasping and gripping, and dexterity exercises such as using cutlery, writing, and drawing.

Electro-physical agents were used daily to reduce pain and spasticity in post-stroke patients with UE dysfunctions: interferential currents, transcutaneous electrical nerve stimulation (TENS), resistive-capacitive radiofrequency therapy, and ultrasound therapy. The patients enrolled in the research only received one of the types of electrotherapy, depending on the needs and the prescription. This treatment did not interfere with the measurements made and presented, which were made on the first day of hospitalization. The electro-physical agents were applied for 10 days.

Because the classical system of scoring muscle strength may be biased according to the examiners’ subjectivity and lack of experience, and due to the plus and minus adjunctions, we designed a system in which the scoring system is assumed to be accurate, and is also easy to use in post-stroke clinical research. In addition, the grading system based on the amplitude of motion offers, as an objective landmark, the mobilization of the segment against gravity, relative to the normal amplitude of motion. The novel scoring system may also provide a better framework for the muscle strength of the whole segment, for muscle strength between 1 and 3 to 4. Previous research [18,22] used a 0.50-unit division between each classical score and was proven to have good inter-rater reliability and precision. Our research provides more specificity by using a 0.25-unit division between the classical MMT scores. Thus, we took into consideration the normal value of the range of motion [37] of every segment tested and divided the value (in degrees) by 4; thus, a more accurate scoring system is proposed. As presented in Table 4, the modified MMT also begin quoting with 0 and ends at 4, within subdivisions of 0.25 units hence, it corresponds to the quarters from the joint’s maximal AROM values.

For example, in elbow flexion, the normal AROM is up to 145°. If it is performed just against gravity, it would be scored as MMT 3. In the classical scoring of MMT, if the evaluated person can perform the elbow flexion against gravity to only 110°, it would be scored as 3− or 2++. If we apply the customized MMT scoring proposed herein, the new scoring value would be 2.75 (Since 145° is considered normal elbow flexion ROM, if the value would be split by 4=, than 36.25° would represent a quarters considering that 36.25 × 3 = 108.75°; therefore, an elbow flexion performed to 110° against gravity would be quoted as 2.75). Regarding the motions performed without gravity, if the patient performed an elbow flexion of 70° in a discarded gravity posture, in the classical scoring system, it would be assessed as 1+ or 2−. Within the newly customized scoring system, the motion performed in a minimized gravity posture at half of maximum AROM would be scored as 1.5.

The MMT evaluation was performed by adjusting Daniel and Worthingham’s method [15] to assess group muscle strength based on the segment direction of motion. For every tested muscle group of the shoulder, elbow, forearm and wrist, the physiotherapist used progressive resistance (after performing 100% ROM) in the opposite direction of the movement maintained by the patient, for a grade higher than 3. The therapist provided resistance by requesting the patient to perform a motor response to the applied pressure and maintain the segment position (static resistance). One hand was placed on the distal segment, while the other hand was placed precisely above the proximal joint, to secure the proximal segment’s stabilization [24]. For the UE, both AROM and MMT were assessed from a sitting, prone, supine, or side-lying position. They were assessed bilaterally.

The AROM assessment was performed manually with a goniometer; for subjects with MMT ≥ 3, both MMT and AROM assessments were performed against gravity.

#### 2.4.1. Shoulder

The shoulder flexors and abductors were assessed against gravity in a sitting position. Stabilization was ensured by positioning the patients with the back or the lateral side near a wall, or by placing the physiotherapist’s hand on the other shoulder to ensure latero-lateral trunk stabilization. For the two motions, against gravity and discarded gravity, we took into consideration shoulder flexion and abduction to 180° (for both AROM and MMT).

For grading < 3, the shoulders abductors were assessed in a supine position, with the UE supported by the therapist.

The shoulder flexors were assessed in a discarded gravity position in a contralateral side-lying position, with the UE supported by the therapist.

For a grade of 3 to 5, the shoulder extensor was assessed in a prone position, and for a grade of 0 to 3, in a contralateral side-lying position, with the upper limb supported by the examiner.

For a 3 to 5 grade, internal rotation and external rotation were assessed in a prone position with the arm at 90° abduction, supported on the table, and with the elbow in 90° flexion. For shoulder internal and external rotations, with discarded gravity, the patient was in a contralateral side-lying position. The arm was in 90° shoulder abduction and 90° elbow flexion, and the forearm was supported by the therapist (we considered that the classical positioning of Daniel and Worthingham’s, in which the patient is in the prone position with an extended elbow and arm at 90° flexion, regarding the post-stroke patients’ patterns of forearm muscle hypertonia, may influence the assessment and compensate the shoulder rotations with forearm motions).

#### 2.4.2. Elbow

Elbow flexors were assessed with the patient in a supine position, with the limb near the side, for grade 3 to 5, whereas for discarded gravity assessment, the patient’s positioning was sitting, with the arm abducted at 90° and supported by the therapist.

The elbow extensor evaluation against gravity was performed in a prone position on the table, with the arm in 90° abduction and the forearm flexed and hanging vertically over the side of the table. For the elbow extensors, in a minimized gravity position (MMT < 3), the assessment was performed in a sitting position, with the arm abducted at 90° and supported by the therapist.

#### 2.4.3. Forearm

The assessment of supination and pronation was performed with the patient in a sitting position, with the shoulder in a neutral position (near the trunk) and the elbow in 90° flexion. For pronation, because the motion is gravity-favored in this position, for MMT = 3 a slight resistance was applied by the therapist. For the evaluation in the minimized gravity position, the patient was in a sitting position, in 90° shoulder flexion and with the arm on the table, while the elbow was set in 90° of flexion and neutral positioning (no pronation or supination (thumb to face)).

#### 2.4.4. Wrist

Wrist flexors were assessed for a grade of 3 to 5 with the patient in a sitting position, with the elbow in 90° of flexion and the forearm in supination. For discarded gravity evaluation of wrist flexors, the patient was in a sitting position, with 90° elbow flexion, forearm and wrist supported by the table, and the forearm neutral (thumb up).

Wrist extensors with MMT ≥ 3 was assessed in a sitting position, with the elbow flexed at 90° and the forearm in pronation. For minimized gravity evaluation, the forearm and wrist were supported by the table, with the forearm neutral.

Radial deviation was assessed in a sitting positioning for MMT ≥ 3, with the elbow at 90°, and the forearm in a neutral positioning (thumb up). To evaluate the muscles that perform radial deviation in minimized gravity, the forearm of the patient (with 90° elbow flexion) was in pronation and supported on the table.

For the ulnar deviation, the patient was in a sitting position, with 90° shoulder flexion, 90° elbow flexion, and the forearm in pronation. For the minimized gravity assessment, the patient was in a sitting position, with 90° elbow flexion, and the forearm and wrist supported by the table.

Finger flexion and extension were assessed from the same positions as those for wrist flexion and extension.

Thumb opposability was assessed with the forearm in a supine position, a neutral wrist, and metacarpophalangeal and interphalangeal proximal in slight flexion (thumb relaxed).

The assessment proposed by us was designed in relation to the amplitude of motion both without the action of gravity and against gravity. For example, although assessments of scapulohumeral joint motions at 90° are presented in Daniel and Worthington and in MRC, in practice, and especially regarding patients with neuromotor impairments, the functional angle of the limbs is more important for the development of ADLs. Thus, it is assumed that a patient with a force 5 on the flexors and abductors of ASH, with limited movement to these values of 90°, cannot perform all the ADLs [38].

Thus, our scoring system is based on a functional assessment with muscle strength values up to grade 4, with the aim of focusing more on recovering the muscle strength necessary for UE functionality, rather than on increasing muscle strength at non-functional angles.

### 2.5. Statistical Analysis

First, the sample size was estimated using G * Power (latest version 3.1.9.7; Heinrich-Heine-Universität Düsseldorf, Düsseldorf, Germany). We assumed the following parameters: effect size f^2^ = 0.35, α = 0.05 and power = 0.95, for the linear multiple regression with an increase in R squared. The a priori sample size calculated for the medium effect size and α = 0.05 was 41 subjects.

To identify the underlying relationships between the measured variables and to determine if our assessment design was reliable, we performed an exploratory factor analysis (EFA) for both UE assessments, pre- and post-intervention, using Principal Axis Factoring (the extraction method) and Quartimax rotation with Kaiser Normalization. The analyzed variables are shown in Table 5 (the muscle group column). To establish if the data passed the assumption of linearity, we explored the correlations matrix, as all the variables should correlate with at least one other variable, with r ≥ 0.3. To verify the sampling adequacy assumptions, we used the Kaiser–Meyer–Olkin (KMO) and the Bartlett sphericity tests, following the KMO > 0.5 and Bartlett *p* < 0.05 parameters, which are considered appropriate values for EFA. The cut-off value for factor loadings was set to 0.30 [39].

Then, we performed confirmatory factor analysis (CFA) using structural equation modeling (SEM) and the unweighted least squares method for estimate calculation [40]. The following indexes of adherence of the model were used: Root Mean Square Residual (RMR), Goodness-of-Fit Index (GFI), Adjusted Goodness-of-Fit Index (AGFI), Normed Fit Index (NFI), and Relative Fit Index (RFI) for baseline comparison, and Parsimonious Normed Fit Index (PNFI) as a parsimony measure. The index values if the model fits are: RFI ≥ 0.9, GFI and NFI ≥ 0.95, AGFI ≥ 0.90, RMR < 0.08, and PNFI ≥ 0.80 [41].

Additionally, we used Cronbach’s Alpha to determine internal consistency. We used the Pearson correlation with AROM, FM, MRS, MAS, and FIM. Therefore, for the CFA we used the data from patients’ hospital admissions, and for the analysis of the other outcome assessments, we used the mean values for every scale before the physical therapy.

For the multiple regression analysis, the stepwise procedure was performed, using the initial assessment values as predictors, and the mean of UE muscle strength value assessed at discharge as the dependent variable, to identify which muscle motions influence the motor function of the UE.

We analyzed the data using the Statistical Package for the Social Sciences (IBM SPSS Statistics for Windows, Version 20.0. Armonk, NY, USA: IBM Corp.) software version 20, and we performed the structural equation modeling (SEM) using Amos (Version 20.0), Chicago: IBM SPSS.

## 3. Results

**Figure 1:**
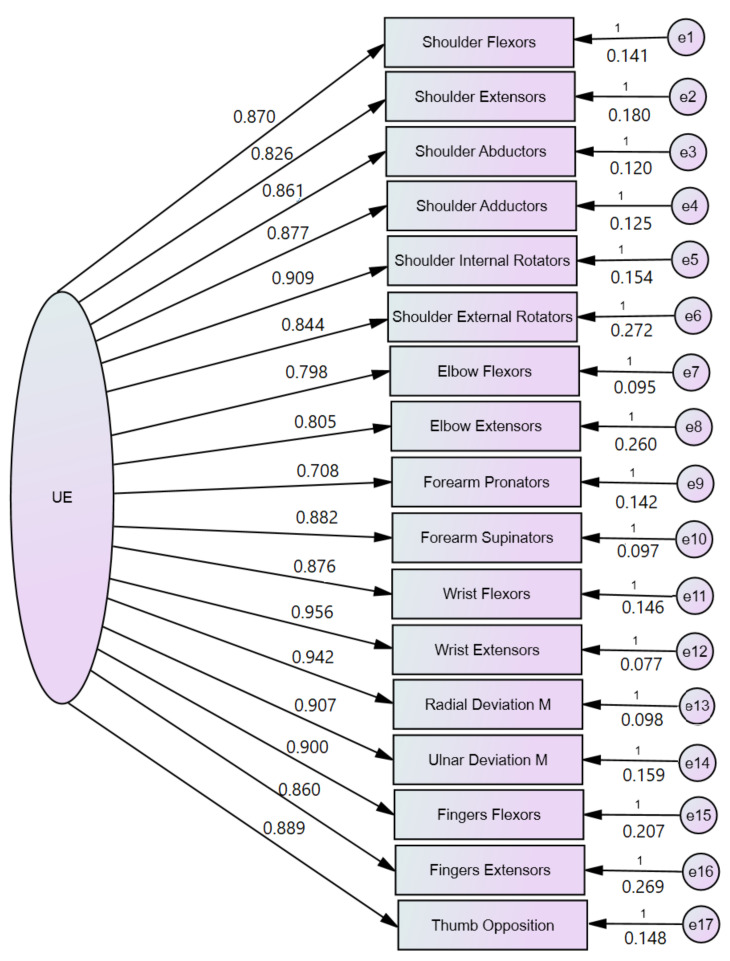
CFA analysis performed with structural equation modeling of the UE Manual Muscle Testing.

Table 5 presents the data collected for EFA and CFA for UE assessment with the post-stroke patient-customized MMT scoring system. The mean and standard deviation (SD) from Table 5 represent the customized scores for the 41 patients enrolled in the research. In Figure 1, we represent the value loadings of standardized regression weights (resulting from SEM) for the upper extremity. The values of the EFA indices for the UE were: KMO = 0.891, chi-square 946.31, *p* < 0.001, 136 df, 1 factor with a 13.065 eigenvalue explaining 76.85% of the variance. The results from EFA suggested that the model fit for CFA may be appropriate for unidimensionality, and the results from CFA confirmed this. The CFA indices for model fit were CMIN = 7.589, 119 degrees of freedom, RMR = 0.036, GFI = 0.996, AGFI = 0.994, NFI = 0.995, RFI = 0.994, PRATIO = 0.875, and PNFI = 0.871, suggesting that the model had a good fit to the SEM regression analysis.

Regarding reliability, the Cronbach Alpha value was 0.920. The Pearson correlation coefficient for AROM was 0.857, *p* < 0.001; that for FMUE was 0.905, *p* < 0.001; that for MRS was −0.608, *p* = 0.010; and that for MAS was −0.677, *p* < 0.001. The correlations were performed on mean values at the first day of hospitalization, for the used scales. The Pearson correlation values suggest that the customized MMT for the UE has a significant correlation with AROM and FMUE, whereas medium negative correlations with MRS and MAS were found.

The multiple linear regression results shown in Table 6 represent the three models that indicate the most important motions to be performed in post-stroke patients’ UEs, in a ten-day post physical therapy program, for better UE motor functioning.

## 4. Discussion

This study aimed to customize the MMT scoring system for the UE assessment in post-stroke patients and to determine whether the proposed approach is scientifically valid for post-stroke patients or research purposes. The confirmatory factor analysis performed through structural equation modeling for the upper extremity had good model fit indicators regarding the unidimensionality of the upper extremity MMT. The results suggest that this adapted form of MMT assessment can be used in the evaluation of post-stroke patients.

Previous studies have drawn different conclusions regarding the use and reliability of this assessment tool [42]. We focused on the reproducibility of the MMT protocol by investigators and found that a more accurate score would provide valuable benefits to both post-stroke patients and physicians, especially in health care settings where post-stroke patients benefit from a short hospital stay. Our research focused on this issue, particularly because chronic post-stroke patients make less rehabilitation progress than those at sub-acute stages [43]. Therefore, we adopted the MMT assessment and redesigned it as a more functional muscle assessment based on the amplitude of movement and the patient’s ability to perform ADLs. However, as shown in the Results section, the average scores for UE do not exceed 4 for muscle strength; therefore, our study cannot be extrapolated to score 4 or 5 for muscle strength. Regarding the upper extremity assessment in post-stroke patients, the evaluation is more complex and requires a broader approach because hand dexterity is involved in the daily motions, and a skilled motion is necessary for writing or professional activities. Many guidelines suggest using different functional scales assessment, such as the Fugl–Meyer Assessment for UE, the Box and Block Test, the Wolf Motor Function Test, the Nine-Hole Peg test, and the Action Research Arm Test, although clinically significant differences were reported in previous research [44,45]. Objective assessment of muscle strength can be performed using electromyography and musculo-skeletal ultrasound [46], and myometry and dynamometry [47,48]. In addition to these methods, only AROM or the validated strength sensor-based technology can assess upper extremity motor function, functionality, or dexterity [49]. The results of our research suggest that the modified MMT for the upper extremities in post-stroke patients is a valuable one-dimension tool; therefore, the results encourage clinicians and researchers to consider all the muscles and motions when performing UE assessments, rather than only several motions or groups of muscles, and thus highlight the importance of evaluating every movement. Moreover, the results of the linear regression were similar to those of the Motricity Index assessment tool used for post-stroke UE evaluation, which enables an assessment of shoulder abduction, elbow flexion, and pinch grip with good reliability [50]. Unlike in the lower extremities, the muscles in the upper extremities that tend to have spasticity seem to present five patterns, with different overactive muscles, such as elbow flexors, shoulder adductors and internal rotators, forearm pronators or supinators, and wrist flexors. Recent research suggested that the most-often encountered pattern is shoulder adduction and internal rotation, flexed elbow, and the forearm and wrist in a neutral position [51]. Our research results from the linear regression show the highest value loadings on the shoulder abductors, wrist extensors, and finger flexion, suggesting that these elements explain a large proportion of UE strength assessed by the customized MMT scoring system, and thus confirm previous research findings on shoulder abductors [7]. Stereotypes of stroke-related inter-joint coordination deficits include the character of the pathological flexor synergy in reaching, compensatory trunk motions for the upper limb limitations, and decreased finger dexterity grasping. The pathological flexor synergy emerges from the co-activation of elbow flexion and shoulder abduction, which manifest in reaching, arm-load-related reductions in the upper limb workspace, and a diminished ability to extend the fingers [52]. Previous research on UE kinematic chain motions for grasping and reaching after spinal cord injury showed modified joint motion patterns for both wrist flexion and extension at C6 and C7 injury sites, with an emphasis on an increased wrist extension ROM, both for grasping and manipulation, and for the lateral grip and whole hand grip [53]. Moreover, recent research focused on robot-assisted training for wrist extensors in post-stroke patients as an essential feature in performing ADLs and synergists in gripping tasks [54]. Thus, the results of our research underline the importance of identifying the movements necessary for the rehabilitation of the upper limbs in post-stroke patients to perform daily activities, with respect to the integration of the extremity in the entire UE open kinematic chain, and particularly for grasping.

The unidimensionality of the adapted MMT system for the upper extremities may be related to the fact that the upper extremity motions require more muscle control and coordination than muscle strength, unlike the lower extremities, where muscle force is necessary to provide support for the standing position and locomotion.

The results of the multiple linear regression applied to identify the motions that are good predictors of upper extremity functionality, namely, shoulder abduction and finger extension, are similar to those in previous studies performed in acute, sub-acute, or chronic settings [55]. Moreover, considering the upper extremity open kinematic chain functionality, for grasping and gripping, motor strength is necessary for the wrist extensors and finger flexors [56]. The results of the multiple linear regression performed in our research may also represent a starting point for further research on how to enhance intensive training by focusing on shoulder abductors, wrist extensors, and finger flexors in neuro-rehabilitation settings, because it seems that UE functionality is influenced by these motions. Recent research suggests the necessity of intensive physiotherapy training in post-stroke patients [57].

Clinical implications regarding our new adapted type of scoring system are valuable in rehabilitation settings where patients having post-stroke neurological impairments can access the public or private health systems for short periods, such as 10 days or two weeks of treatment, every 3 or 4 months, as per the Romanian National Health Insurance system. As we described earlier, neuromotor changes in muscle activity are less sensitive in short-term assessments; therefore, our proposed model can be successfully used in rehabilitation settings where patients are discharged after two weeks. Unfortunately, in Romania, rehabilitation programs for stroke survivors tend to be unplanned, irregular, and difficult to obtain, resulting in patients not recovering significantly, or not benefiting from periods of rehabilitation progression (inpatients) and regression (discharged home).

In addition, the customized MMT used in our research can be less time consuming in rehabilitation settings, for grade 3 to 4 muscle strength, because AROM assessment is performed against gravity. Thus, based on the AROM quarter split, the physiotherapist can estimate or appreciate with better accuracy the muscle strength, especially for grade 3. Moreover, if performed on the entire UE, this assessment can provide a mean score that can be easily followed in future hospital admissions, because shoulder abduction, wrist extension, and finger flexion values are also meaningful indices, according to the linear regression results.

Although new technologies such as robots, virtual reality, or other sensor-based technologies have been developed for rehabilitation in post-stroke or central nervous system impairments with motion disorders, each device is used with its own type of evaluation [58,59,60,61,62,63]. Due to the heterogeneity of these devices and the types of assessment provided, it is difficult for researchers and clinicians to use them properly and include them in daily practice. It is also difficult to validate them via research because scientific validation requires multiple analyses, including CFA, and different types of assessment tools are integrated into many devices. The need for good assessment tools, scales, or technologies is highly demanded in rehabilitation, especially in neuro-rehabilitation, due to new technology growth, and because of the increase in the healthcare, social, and familial burdens caused by stroke sequelae [64]. Furthermore, the customized MMT scoring provided in our research is a promising tool for clinicians and physiotherapists, especially in neuro-rehabilitation assessment, since the functions are restored over an extended period, and relatively poor variations may be acquired and established in short periods (a few weeks or a month). For example, a patient with a classic shoulder flexion score of 3 minus (−) on the first day of hospitalization can initiate a movement against gravity. If, at discharge, after ten days of therapy, the patient is still unable to mobilize shoulder flexion to 180°, against gravity, it would also be scored as a 3 minus (−). Using the new scoring scale proposed in our research, if the patient was able to perform shoulder flexion of 45° against gravity on the first day of hospital admission, it would be rated 2.25. If he was able to perform the same movement of 140° against gravity at discharge, this would be scored as 2.75. Thus, the adapted MMT scoring system customized for post-stoke patients may provide meaningful feedback to neuro-rehabilitation specialists for the enhancement of assessment performance and specific goal and timeframe setting.

### Limitations

One of the most important limitations of this study is the fact that this type of assessment was performed only in patients who passed the sub-acute phase of post-stroke and had mild deficits. However, we must emphasize that the majority of Romanian post-stroke patients do not benefit from rehabilitation unless they are admitted to a rehabilitation clinic for 2 weeks or are able to use outpatient rehabilitation services (public or private), at most 4 times per year for a period of 2 weeks, covered by health insurance. Thus, considering the discharge periods, the improvement in motor function and post-rehabilitation functionality is lost during the period when most patients do not perform physiotherapy programs. Thus, future studies should be performed in the functional sub-acute stage of the post-stroke patient, but with a confirmatory aim, because the most significant changes in neuro-rehabilitation are usually observed in these stages, and considering if the patients benefit from physiotherapy programs between hospital admissions.

An essential feature is the lack of assessment with a technologically certified device that can measure muscle strength, such as a dynamometer. Therefore, the proposed model for MMT scoring could only be compared with functional assessment scales such as the Fugl–Meyer assessment, because some motion assessments were modified based on the functional angle, for daily activities. Moreover, the Fugl–Meyer assessment has proven to be a reliable tool in the evaluation of post-stroke patients. Another major limitation concerns inter-rater and intra-rater reliability because our research did not include this analysis; however, the physiotherapists who performed the assessment did not know which patients were enrolled in the research, and the data presented in this paper were based mainly on the assessment performed on the first day of hospital admission. Thus, although the results of our study can only be considered relevant to the conditions under which the research was conducted and the tools used, they can be a basis for future research.

## 5. Conclusions

The post-stroke patient-customized MMT scoring system proposed in our research may be a valuable tool in post-stroke patients’ disease management. The proposed system can be used to both assess the UE motor function and strength, and to help in setting rehabilitation goals and in outlining physiotherapy programs, which are of outmost importance for the lives of post-stroke patients. This adapted MMT scoring system, according to the results presented herein, more clearly emphasizes rehabilitation progress with even slight variations in motor function, given the short periods in which post-stroke patients can benefit from physiotherapy programs. Thus, medical practitioners have better insights into the way that the short-term goals of rehabilitation programs are met. Further research is needed to establish the validity and usability of the modified MMT, including inter-rater and intra-tester reliability. Moreover, comparisons should be made with objective muscle strength measurements, and in other rehabilitation areas, such as musculoskeletal disorders or rheumatic diseases.

## Figures and Tables

**Table 1:** The classical Manual Muscle Testing scoring system.

Grade	Description	Criteria
0	No contraction	No contraction can be felt in the muscle
1	Trace muscle contraction	Muscle contraction can be felt on palpation but without motion
2	Poor muscle contraction	Muscle contraction and motion of the segment in a gravity discarded position (gravity minimized)
3	Muscle contraction	Full motion of the segment against gravity
4	Good muscle contraction	Full motion of the segment against gravity and moderate resistance
5	Normal muscle contraction	Full motion of the segment against gravity and maximal resistance

**Table 2:** Patient’s characteristics.

Characteristic	UE (n = 41)
Age (mean/SD)	63.05/6.82
Gender (n/%)	
Male	20/48.78%
Female	21/51.22%
Time since stroke (years) (mean/SD)	1.91/1.00
Affected side (n)	
Left	16/37.50%
Right	25/62.50%

**Table 3:** Outcome measures for UE assessments in post-stroke patients.

Type of Assessment	Aim	Instrument Characteristic
Functional Independence Measure (FIM)	Identifying and assessing patients’ independence in performing activities of daily living (ADLs).	A reliable and valid instrument with a good construct validity in post-stroke patients [26]
Modified Rankin Scale (MRS)	Assessing stroke severity and patients’ degree of disability.	Concurrent validity. A robust predictor of prognosis and validity, with modest inter-rater reliability on ICC [27,28]
Modified Ashworth Scale (MAS)	Measuring spasticity.	Satisfactory inter-and intra-rater agreement. Moderate reliability, with better reliability in UE than in LE assessment [29,30]
Active Range of Motion (AROM)	Assessing the range of motion of human body joints.	Excellent test-retest reliability score. Can predict UE function at three months post-stroke [31,32]
Manual Muscle Testing (MMT)	Assessing muscle strength.	Validity needs to be re-evaluated [24]
Fugl–Meyer Upper Extremity Assessment (FMUE)	Assessing the UE motor functioning, sensation, and coordination.	Good reliability, construct validity, inter-rater and intra-rater reliability [33,34,35]

**Table 4:** Adapted MMT based on AROM.

AROM Split Value	MMT Value	MMT Technique
100% (4/4) * AROM max	4	Moderate resistance
75% (3/4) * AROM max	3.75	* Slight resistance against Gravity
50% (2/4) * AROM max	3.50	Slight resistance against Gravity
25% (1/4) * AROM max	3.25	Slight resistance against Gravity
100% (4/4) * AROM max	3	Against gravity
75% (3/4) * AROM max	2.75	Against gravity
50% (2/4) * AROM max	2.50	Against gravity
25% (1/4) * AROM max	2.25	Against gravity
100% (4/4) * AROM max	2	Minimized gravity
75% (3/4) * AROM max	1.75	Minimized gravity
50% (2/4) * AROM max	1.5	Minimized gravity
25% (1/4) * AROM max	1.25	Minimized gravity
0–25% * AROM max	1	Minimized gravity
0% * AROM max	0	Minimized

* Moderate resistance and slight resistance against gravity is the same, except for the intensity of the resistance provided by the physiotherapist.

**Table 5:** Factor loadings for UE MMT assessment with EFA and CFA.

Muscle Group	Mean ± SD (n = 41)	Communalities Extraction (EFA)	Factor Matrix Loading (EFA)	Standardized Regression Weights SEM (CFA)
Shoulder Flexors	3.00 ± 0.77	0.791	0.889	0.870
Shoulder Extensors	3.03 ± 0.76	0.706	0.840	0.826
Shoulder Abductors	2.92 ± 0.69	0.779	0.883	0.861
Shoulder Adductors	3.08 ± 0.75	0.787	0.887	0.877
Shoulder Int. Rotators	2.72 ± 0.95	0.807	0.898	0.909
Shoulder Ext. Rotators	2.54 ± 0.98	0.737	0.858	0.844
Elbow Flexors	3.29 ± 0.52	0.667	0.816	0.798
Elbow Extensors	3.17 ± 0.87	0.648	0.805	0.805
Forearm Pronators	3.12 ± 0.54	0.521	0.722	0.708
Forearm Supinators	2.79 ± 0.67	0.791	0.889	0.882
Wrist Flexors	2.80 ± 0.80	0.762	0.873	0.876
Wrist Extensors	2.58 ± 0.95	0.898	0.948	0.956
Radial Deviation M	2.39 ± 0.94	0.874	0.935	0.942
Ulnar Deviation m	2.56 ± 0.96	0.803	0.896	0.907
Fingers II-V Flexors	2.98 ± 1.06	0.785	0.875	0.900
Fingers II-V Extensors	2.71 ± 1.03	0.710	0.842	0.860
Thumb opposition M	2.76 ± 0.85	0.763	0.874	0.889

**Table 6:** Linear regression for assessing the UE segments.

Model	Unstandardized Coefficients (B)	Standardized Coefficient β	*p*	R^2^
1. (Constant)	1.342		0.000	0.822
Wrist extensors	0.734	0.909	0.000	
2. (Constant)	0.647		0.001	0.898
Wrist Extensors +	0.467	0.578	0.000	
Shoulder abductors	0.474	0.425	0.000	
3. (Constant)	0.578		0.001	0.911
Wrist Extensors +	0.285	0.353	0.002	
Shoulder abductors+	0.449	0.402	0.000	
Fingers Flexors	0.205	0.282	0.005	

## Data Availability

Data of this paper can be accessed by request, through a standard application procedure according to local health data-sharing regulation.
